# Serum Monocyte Chemoattractant Protein-1 in Pancreatic Cancer

**DOI:** 10.1155/2011/518394

**Published:** 2011-10-01

**Authors:** Jennifer Sullivan, Qiaoke Gong, Terry Hyslop, Harish Lavu, Galina Chipitsyna, Charles J. Yeo, Hwyda A. Arafat

**Affiliations:** ^1^Department of Surgery and Jefferson Pancreatic, Biliary and Related Cancer Center, Thomas Jefferson University, Philadelphia, PA 19107, USA; ^2^Department of Pharmacology and Experimental Therapeutics, Thomas Jefferson University, Philadelphia, PA 1015, USA; ^3^Department of Pathology Anatomy and Cell Biology, Thomas Jefferson University, Philadelphia, PA 1015, USA

## Abstract

*Background/Aims.* Pancreatic ductal adenocarcinoma (PDA) has etiological association with chronic inflammation. Elevated circulating levels of inflammatory mediators, such as monocyte chemoattractant protein-1 (MCP-1), are found in obese individuals. We hypothesized that serum MCP-1 levels are elevated in obese PDA patients. *Methods.* ELISA was used to analyze MCP-1 serum levels in PDA (*n* = 62) and intraductal papillary mucinous neoplasms (IPMN) (*n* = 27). Recursive partitioning statistical analysis investigated the relationship between log MCP-1 and clinicopathological parameters. *Results.* Log MCP-1 values were significantly (*P* < 0.05) elevated in patients with BMI ≥ 37.5. In patients with BMI < 37.5, average log MCP-1 values were significantly elevated in PDA patients when compared to IPMN patients. Within the IPMN group, higher log MCP-1 levels correlated with increased age. Recursive partitioning analysis of IPMN versus PDA revealed a strategy of predicting characteristics of patients who are more likely to have cancer. This strategy utilizes log MCP-1 as the primary factor and also utilizes smoking status, gender, and age. *Conclusion.* MCP-1 is a promising biomarker in pancreatic cancer. The potential of using MCP-1 to distinguish PDA from IPMN patients must be studied in larger populations to validate and demonstrate its eventual clinical utility.

## 1. Introduction

With an estimated 35, 420 deaths in 2008, pancreatic cancer is the fourth leading cause of cancer death in the United States [1]. Pancreatic cancer has an overall five-year survival rate of only 4%, as fewer than 10% of patients' tumors are confined to the pancreas at the time of diagnosis. In most cases, the cancer has progressed to the point where surgical resection is impossible. In a disease that is still considered most often incurable, there remains a need for new strategies for prevention and novel methods for early diagnosis.

One factor that is believed to have an important role in the development of pancreatic cancer is obesity. Obesity is defined as a body mass index (BMI) > 30, and in the United States, more than 30% of the population is classified as obese [2]. Several studies have shown that the adipose tissue is an active source of inflammatory mediators, suggesting that obesity causes a chronic, low-level inflammatory state [3]. This is thought to contribute to the development of many of the comorbidities found in obese patients, such as atherosclerosis, diabetes, and cancer [4, 5]. This concept is supported by studies that have observed altered chemokine levels and deceased cancer mortality rates with weight loss or in morbidly obese patients that have undergone bariatric surgery [6, 7]. The mediators of chronic inflammation, such as cytokines, free oxygen radicals, and chemokines, can cause cellular injury, DNA damage, and increased proliferation, creating a microenvironment in which carcinogenesis is favored [8, 9].

One of the key chemokines involved in the initiation of inflammation is monocyte chemoattractant protein-1 (MCP-1). MCP-1 triggers chemotaxis and transendothelial migration of monocytes by interacting with their membrane CC (have two adjacent cysteine amino acids near their amino terminus) chemokine receptor 2 (CCR2) [10, 11]. In squamous cell carcinoma of the esophagus, MCP-1 expression in cancer cells was correlated with venous invasion, distant metastasis, and lymph node metastasis [12]. In addition, MCP-1 was demonstrated to act as a potent chemotactic factor for myeloma cells [13, 14]. MCP-1 gene transfer has been shown to enhance the metastatic potential of cancer cells with increased neovascularization [15]. To date, most studies in cancer have focused on MCP-1 tissue expression while only a few have investigated the clinical utility of its serum level measurements. With the exception of a few publications on cervical and ovarian cancers [16, 17], which showed a correlation between MCP-1 systemic levels and cancer progression, no studies have evaluated MCP-1 serum levels in correlation with PDA risk factors, such as obesity. This, in association with other known pancreatic cancer risk factors, such as age and smoking, may contribute to predicting which patient population is more at risk to develop pancreatic cancer [18].

In this study, we investigated the relationship between increased body weight and BMI in pancreatic cancer patients and the circulating levels of MCP-1. We also evaluated whether MCP-1 serum levels could serve as differentiation marker for benign and malignant lesions. In this respect we examined MCP-1 serum levels in PDA patients and in patients with benign IPMNs.

## 2. Materials and Methods

### 2.1. Patients

This retrospective study included 89 patients with pancreatic lesions who underwent pancreatic resection. Blood was collected by venous puncture prior to surgery from patients who underwent surgical resection at Thomas Jefferson University Hospital between 2005 and 2008. Serum samples were prepared and stored at −80°C until analyzed. Sixty-two patients had pathologically confirmed invasive PDA, and twenty-seven had intraductal papillary mucinous neoplasms (IPMNs). Clinical data were obtained from Thomas Jefferson University Hospital electronic medical records and from patients charts. All patients signed an appropriate consent form. The study was approved by the Institutional Review Board at Thomas Jefferson University. Clinical data, tumor characteristics, and AJCC staging of these patients are shown in Table 1.

### 2.2. Methods

Serum concentrations of MCP-1 were measured using commercially available human MCP-1-specific enzyme-linked immunosorbent assays (ELISAs) (R&D Systems, Minneapolis, MN) according to the manufacturer's protocol. Spectrophotometric evaluation of MCP-1 levels was analyzed by Synergy HT multidetection Microplate reader (BioTeck, Winooski, VT).

### 2.3. Statistical Analysis

Multivariable logistic regression analysis was used to determine the association of clinical factors and the protein MCP-1 with pancreatic cancer status. MCP-1 protein expression was analyzed first as raw levels then in a logarithmic scale, in order to achieve a symmetric distribution. An assessment of sensitivity and specificity of the multivariable logistic regression model was completed using receiver-operating characteristic (ROC) methods. As an alternative approach, recursive partitioning analysis was used in two ways, first to demonstrate the relationship of MCP-1 with demographic and clinical characteristics, and second to demonstrate the overall differences between demographic, clinical, and MCP-1 characteristics of patients with pancreatic cancer and patients with benign pancreatic lesions (IPMNs). Sensitivity and specificity of the resulting algorithm to determine cancer versus benign pathology was completed using the standard definitions of these measures. A linear mixed model was used to compare mean values of MCP-1 across defined subgroups from the recursive partitioning model. Linear contrasts of the least squares means were used to compare across recursive-partitioning defined subgroups. No adjustment for multiple comparisons was made to the *P* values. Probabilities ≤ 0.05 were considered significant. Analysis was completed using R version 2.9.2 and SAS version 9.2 (SAS; Campus Drive, Cary, NC).

## 3. Results

### 3.1. Patient Characteristics

The patients' data and clinicopathological parameters are summarized in Table 1. The average age at diagnosis was 64.7 years (range 41 to 80 years) for PDA patients and 66.6 years (44–81 years) for IPMNs patients. The average duration of followup was 15.2 months (range, 1.5–31 months). BMI was calculated as weight in kilograms divided by the square height in meters, based on the patient's preoperative admission data. The National Heart, Lung, and Blood Institute's definition for overweight and obesity was used to classify the patients by their weight. A BMI < 18.5 was classified as underweight; a BMI between 18.5 and 24.9 was classified as normal weight; a BMI between 25.0 and 29.9 was classified as overweight; a BMI > 30 was classified as obese. The mean BMI for all patients in this study was 26.6 kg/m^2^. Values of MCP-1 in pg/mL in each group are seen in Tables 1 and 2.

### 3.2. MCP-1 Serum Levels in Obese Patients

Values for MCP-1 were logarithmically transformed prior to significance testing, as their distributions were skewed. Recursive partitioning analysis of log MCP-1 indicates that log MCP-1 values are elevated in patients with BMI ≥ 37.5 (average log MCP-1 = 6.68). In patients with BMI < 37.5, elevated MCP-1 levels are seen in patients with cancer (average log MCP-1 = 4.94). In patients with benign lesions, log MCP-1 values are elevated with age (age < 55, average log MCP-1 = 3.59; age ≥ 55, average log MCP-1 = 4.81). Using contrasts from the linear model, these mean differences were tested, with results presented in Table 3.

### 3.3. MCP-1 Serum Levels, Patient Clinicopathological Parameters, and the Presence of PDA Compared with Benign IPMN

**Figure 1:**
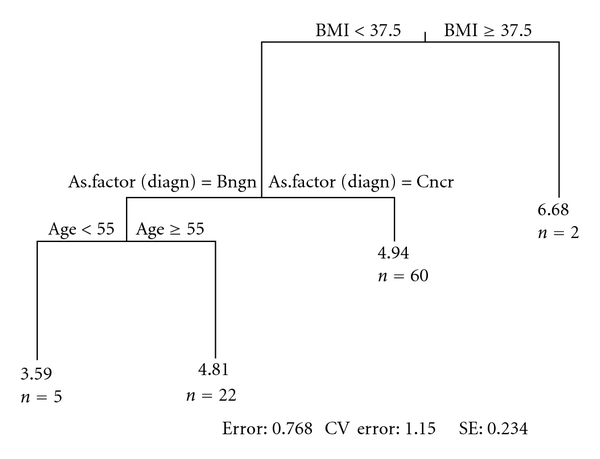
Multivariate logistic regression analysis was used to determine the association of clinical factors and the protein MCP-1 with pancreatic cancer status. An assessment of sensitivity and specificity of this model was completed using receiver-operating characteristic (ROC) methods. Recursive partitioning analysis was used to demonstrate the relationship of log MCP-1 with demographic and clinical characteristics. The lowest levels of log MCP-1 were obtained in benign nonobese patients under the age of 55 years (3.59). Benign nonobese patients aged 55 years and over had average log MCP-1 levels of 4.81, and nonobese cancer patients had log MCP-1 levels of 4.94. Obese patients, regardless of cancer status, had the highest level of log MCP-1 (6.68).

Recursive partitioning analysis of benign versus malignant pathology reveals a strategy of predicting characteristics of patients who are more likely to have cancer. This strategy utilizes log MCP-1 as the primary factor and also utilizes smoking status, gender, and age. The algorithm achieves 95% sensitivity and 59% specificity. As seen in Figure 1, recursive partitioning demonstrates that the lowest levels of log MCP-1 were obtained in benign nonobese patients under the age of 55 (log MCP-1 = 3.59). Benign nonobese patients 55 years old and over had average log MCP-1 levels of 4.81, and nonobese cancer patients had MCP-1 level of 4.94. Obese patients, regardless of cancer status, had the highest level of log MCP-1 (6.68).

### 3.4. MCP-1 Serum Levels and Cancer Predictability

**Figure 2:**
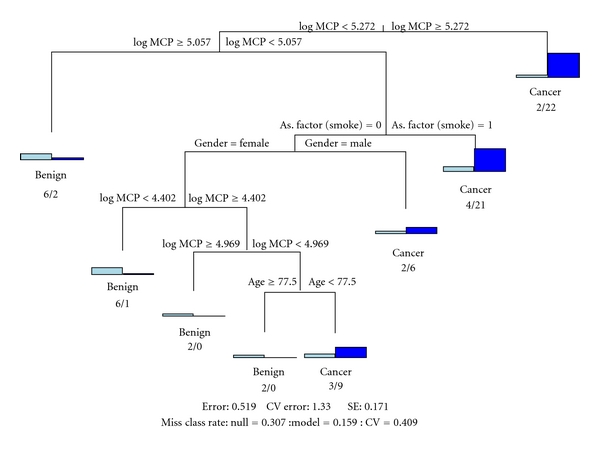
Recursive partitioning analysis was used to demonstrate the overall differences between demographic, clinical, and log MCP-1 characteristics of patients with PDA and patients with benign IPMN. Sensitivity and specificity of the resulting algorithm to determine cancer versus benign patients was completed using the standard definitions of these measures. Word at end of node demonstrates that the algorithm predicts either Benign or Cancer for the subgroup and graph then represents how many Benign/Cancer patients are in each subgroup. Standard logistic regression analysis utilizing log MCP-1 could not attain this level of sensitivity and specificity combined, rather it could achieve high values of one of these metrics at the expense of the other.

Log MCP-1 in multivariate logistic regression analysis controlling for gender, age, BMI, and quartiles of log MCP-1 was completed. As seen in Figure 2, patients within the highest quartile of log MCP-1 were 6.2 times more likely to be pancreatic cancer patients when compared to patients within the lowest quartile of log MCP-1 (adjusted odds ratio 6.2, 95% confidence interval [1.1, 37.4], *P* = 0.04). The ROC curve resulting from this model could not identify a combination that obtained both high sensitivity and high specificity.

## 4. Discussion

With the rising epidemic of obesity, several studies have revealed that the chronic inflammatory changes associated with obesity may explain some comorbidities in obese patients and even the increased incidence of cancer in this patient population [19, 20]. Cytokines and chemokines and their receptors have been analyzed in cancer patients and animal models to attempt to elucidate this link. 

Chemokines form a superfamily of small, inducible, proinflammatory chemotactic cytokines. These proteins have been reported to be involved in a variety of immune and inflammatory responses, acting primarily as chemoattractants and activators of specific leukocytes. MCP-1 is a member of the C-C subfamily of chemokines and is expressed upon stimulation in a wide variety of cells, such as mononuclear leukocytes, fibroblasts, endothelial and epithelial cells, smooth muscle cells, melanocytes, and various tumor cells [21]. MCP-1 is a key element of the immunological response to malignant growth, mainly via attraction and activation of tumor-associated macrophages [22]. The presence of high tumor levels of MCP-1 has been reported in several malignancies in relation to tumor progression [11–15]. However, no previous studies have examined serum levels of MCP-1 in correlation with pancreatic cancer risk factors. To our knowledge, this is the first report (a) demonstrating a positive correlation between the level of a particular chemokine and the subject BMI as a risk factor for PDA and (b) revealing the potential validity of MCP-1 as a differentiation marker for benign and malignant lesions.

Our data show that although raw value of MCP-1 did not show significance when correlated with the different clinicopathological parameters in PDA (Table 1) or IPMN patients groups, patients with BMI ≥ 37.5 averaged higher log MCP-1 values than patients with a BMI < 37.5 when using recursive partitioning analysis. These data support the findings of other studies showing correlation of high circulating MCP-1 levels in obese individuals [23, 24] and in type 2 diabetes [25]. However, previous studies have shown that serum levels of MCP-1 are decreased after weight loss in obese healthy individuals [24, 25]. Due to the late presentation of pancreatic cancer, many patients may have already lost a considerable amount of weight by the time they have presented for surgical intervention. Therefore, our patients' serum MCP-1 levels during their peak BMI may have decreased by the time of their pancreatic resection. Thus, based on our present data, we cannot conclude that weight loss due to PDA itself may be associated with reduced MCP-1 levels in PDA patients and future studies are needed to test this hypothesis.

Our data also show that, in the less obese patients, there was high association between elevated BMI and increased log MCP-1 levels in PDA patients compared to the nonsignificant association in patients with benign IPMNs. In those patients with benign lesions, higher values were seen with age >55. These findings might be independent of the benign lesion itself, since previous studies have reported increased circulating levels of MCP-1 with advanced age [26].

 Previous studies have shown the validity of MCP-1 as a differentiation marker between benign and malignant lesions in gliomas [27] and ovarian cancer patients [28]. In our studies, using multivariate logistic regression analysis we examined MCP-1 in relation to other clinical characteristics and risk factors to evaluate which groups had an increased rate of PDA. First, we found that patients within the highest quartile of log MCP-1 were 6.2 times more likely to have pancreatic cancer than those patients within the lowest log MCP-1. In the patients with the lower quartile of log MCP-1, increased rate of cancer was seen in those with known risk factors for pancreatic cancer, such as smoking or male sex. It remains to be determined, however, whether MCP-1 could serve as a comparable marker independent of the established marker for pancreatic cancer CA 19–9. These studies that are currently ongoing in our laboratory are important to document the validity of MCP-1 as a possible candidate in the ongoing efforts to find adequate combined screening systems for high risk patients and to monitor the prognosis of PDA patients following surgical resection.

A single study has been published so far regarding the prognostic value of MCP-1 serum levels in PDA. Christiansen et al. (2005) [27] reported that higher MCP-1 serum levels correlate with increased macrophage infiltration of pancreatic cancer tissue and with a favorable prognosis and overall survival. However, it has also been reported that monocyte/macrophage infiltration of malignant tissue may promote tumor growth by producing various chemokines [8, 9]. In our series, MCP-1 serum levels showed no clear influence on the patients' prognosis regarding disease-free and overall survival. Although the cellular sources of circulating MCP-1 are not determined, our data indicate that MCP-1 is a promising biomarker that might play a functional role in the natural history of PDA, especially in obese patients. 

### 4.1. Study Limitations

Since this was a pilot study, we focused only on our patients' population. A larger study that includes age and weight-matched healthy volunteers and a larger number of patients is currently under preparation. Comparative data on CA-19–9 and other markers of inflammation, such as C-reactive protein, could be extremely valuable to determine how MCP-1 levels compare to other factors. Studies to address these deficiencies are currently ongoing in our laboratory.

Because the findings of this study are limited to surgically resected patients with PDA or IPMN, the temporal relationship between MCP-1 level changes in the serum and the progression of PDA remains unknown. The absence of significant correlations between clinicopathological parameters and the circulating MCP-1 levels should be interpreted with caution. The sample size was relatively small, and our follow up period was relatively short for association studies and may have mitigated against demonstrating statistical associations. Moreover, further study of this protein in different benign and unresectable malignant lesions, to understand the nature of the relationship of its elevation with age and with highest levels of obesity, will assist with the full development of the potential clinical usage of this marker.

## 5. Conclusion

The promise demonstrated using MCP-1 plus other patient characteristics to distinguish patients with benign versus malignant disease must be studied in larger populations to both validate and demonstrate their eventual clinical utility.

## Figures and Tables

**Table 1:** Clinicopathological characteristics of PDA patients and MCP-1 levels.

	PDA *n* = 62	%	MCP-1 (pg/mL) mean ± SEM	*P* value	Survival at 12 months	*P* value
*Average age (yrs)*	64.7					
<60 (mean 52.9)	20		211.1 ± 68.37	0.12	83%	0.9
>60 (mean 70.2)	42		232.91 ± 51.69		74%	
M	28	45	209.1 ± 50.5	0.9	85%	0.5
F	34	55	239.7 ± 62.9		67.9%	
*BMI*						
Normal weight (<25)	20	32	174.3 ± 30.7		77.7%	
Overweight (25–29.9)	28	45	227.8 ± 55.3	0.09	75.7%	0.08
Obese (>30)	14	23	277.5 ± 141		71.3%	
*AJCC stage*						
I	5	8	69.12 ± 22.04		80%	
II	46	74	228.43 ± 35.5	0.62	75%	0.4
III-IV	11	18	104.52 ± 15.6		62%	
*Grade*						
G1	8	13	280.15 ± 119.6		83%	
G2	37	60	186.99 ±102.63	0.34	80%	0.5
G3-G4	17	27	302.63 ± 38.82		65%	
*Lymph nodes*						
N0	7	11	106.08 ± 30.37		83%	
N1-N2	16	26	155.04 ± 26.77		80%	
N3-N5	17	27	321.82 ± 85.19	0.07	63%	0.06
>N6	22	36	241.16 ± 91.066		50%	

**Table 2:** Clinicopathological characteristics of IPMN patients and MCP-1 levels.

	IPMN *N* = 27	%	MCP-1 (pg/mL) mean ± SEM	*P* value
*Average age (yrs)*	66.6			
<60 (mean 53.3)	9	33	87.9±17.6	0.09
>60 (mean 72.8)	18	67	142.7± 17.7	
M	7	26	152.9 ± 21.9	0.12
F	20	74	113.9 ± 14.3	
*BMI*				
Normal weight (<25)	10	37	80 ± 14.1	
Overweight (25–29.9)	10	37	150 ± 17.7	0.34
Obese (>30)	7	26	132 ± 30	

**Table 3:** Subgroups of levels of log MCP-1 as defined by recursive partitioning. Data in diagonal cells represents least squares mean of the specified subgroup, while unadjusted *P* values are provided in the off-diagonal cells.

	BMI < 37.5, diagnosis: benign, age < 55	CI	BMI < 37.5, diagnosis: benign, age ≥ 55	CI	BMI < 37.5, diagnosis: cancer	CI	BMI ≥ 37.5, diagnosis: cancer	CI
BMI < 37.5, diagnosis: benign, age < 55	3.59	2.9–4.3						

BMI < 37.5, diagnosis: benign, age ≥ 55	*P* = 0.003		4.81	4.5–5.1				

BMI < 37.5, diagnosis: cancer	*P* < 0.001		*P* = 0.46		4.94	4.8–5.1		

BMI ≥ 37.5, diagnosis: cancer	*P* < 0.001		*P* < 0.001		*P* = 0.001		6.68	5.7–7.7
